# The effects of combined exercise intervention based on Internet and social media software for postoperative patients with breast cancer: study protocol for a randomized controlled trial

**DOI:** 10.1186/s13063-018-2857-3

**Published:** 2018-09-06

**Authors:** Dong Xiaosheng, Yi Xiangren, Huang Shuyuan, Gao Dezong, Chao Mengyao, Ding Meng

**Affiliations:** 1 College of Physical Education, Shandong Normal University, Jinan, 250014 China; 2 College of Physical Education, Shandong University, Jinan, 250011 China; 3 The Department of Breast Surgery, the Second Hospital of Shandong University, Jinan, 250033 China

**Keywords:** Internet and social media software, Combined exercise, Breast cancer, Quality of life, SF-36, Physical fitness

## Abstract

**Background:**

Many randomized controlled trials have investigated the effects of exercise on the rehabilitation of patients with breast cancer. However, the exercise forms used in most previous studies were monotonous. Therefore, we designed a protocol to estimate the effects of combined exercise intervention using Internet and social media software on the rehabilitation of postoperative patients with BC.

**Methods/Design:**

This study protocol is a randomized control trial with an intervention time of 12 weeks. After completing baseline questionnaire and physical fitness tests, the participants are randomized to the study group or the control group. Procedure contents of exercise intervention in the study group include: via phone step-recording app, ask the individuals to complete the target number of steps within a specified period of exercise, four times per week; face-to-face remote video guidance of individuals on muscle training, three times per week; common knowledge of physical exercise BC rehabilitation will be pushed regularly by social media apps every day. The control group will receive normal treatment and rehabilitation according to daily specifications of the hospital. The primary outcome will be the quality of life. The secondary outcomes are physical fitness and social cognitive indicators.

**Discussion:**

This study is a clinical trial to estimate the effects of combined exercise intervention based on the Internet and social media software for postoperative patients with breast cancer (BC). If expected results are achieved in this study, measures and methods of BC rehabilitation will be enriched.

**Trial registration:**

Chinese Clinical Trial Register, ChiCTR-IPR-17012368. Registered on 14 August 2017.

**Electronic supplementary material:**

The online version of this article (10.1186/s13063-018-2857-3) contains supplementary material, which is available to authorized users.

## Background

Breast cancer (BC) is a type of malignant cancer that severely harms women’s health worldwide and is the most common cancer in women in both developed and developing countries. In developing countries, the incidence of breast cancer is rising due to increased life expectancy, the expansion of urbanization, and the adoption of a western lifestyle [1]. The most common manifestations in postoperative BC patients are mental fatigue, anxiety, and even depression [2]. The functions of the upper extremities may be affected, which also leads to limited physical activity. Long-term reduced physical activity will cause decreased adult bone mineral density and decreased muscle and cardiopneumatic endurance, which will severely affect emotions and quality of life (QOL) [3].

It has been demonstrated in modern medical studies, both domestic and abroad, as well as clinical practice that sport activities may improve muscle strength and cardiopneumatic endurance in BC patients during BC rehabilitation and further improve their emotions and QOL [3–9]. In addition, some studies have shown that resistance training has a certain effect on the improvement of postoperative complications of lymphedema [10–12]. Therefore, it has been recommended that both resistance training and aerobic exercise be included in the postoperative BC rehabilitation plan [3]. Qigong is one type of traditional Chinese exercise which is a combination of physical activity, breathing exercises, and psychological adjustment that has long been applied in health promotion and the treatment of various chronic diseases [13–16]. Theoretically, fitness Qigong may improve postoperative QOL, physical fitness, and psychological status of BC patients. With the universalization and extensive applications of the Internet, exercise intervention and guidance via phone social media apps and remote video to BC patients becomes possible. Currently, some related studies have been carried out [17–19], but most of the previous studies express the same problem that the means of exercise intervention is overly simple, which may impact the effect of the intervention.

Therefore, we have designed a combined exercise intervention protocol applying multiple Internet social media and plan to evaluate the effectiveness of the protocol by a clinical trial using a high-quality methodology: to conduct a randomized controlled trial (RCT) to estimate the effects of combined exercise intervention based on the Internet and social media software for postoperative patients with BC.

## Methods and design

### Study design

**Fig. 1:**
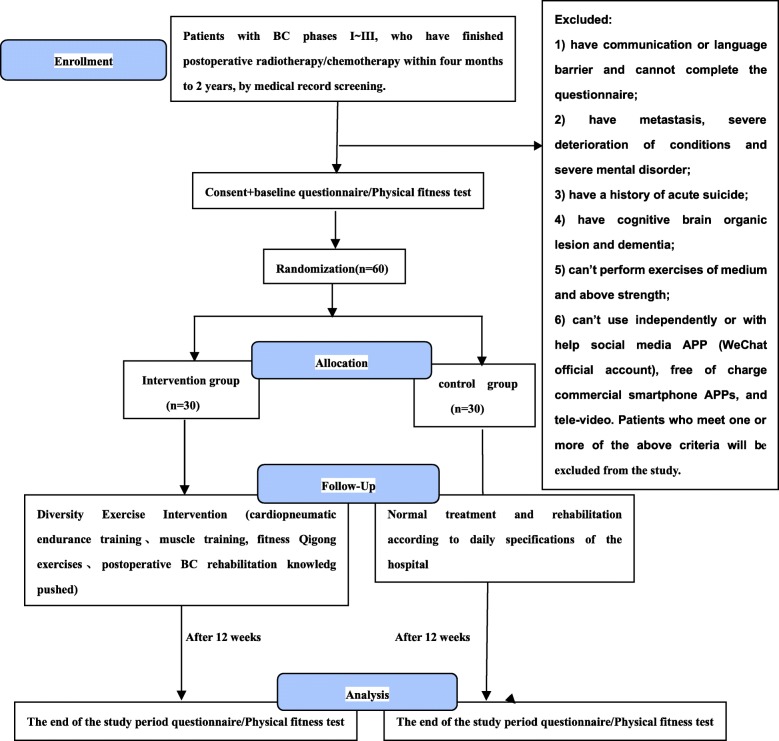
The flow chart of the trial design

This study is a RCT with an intervention time of 12 weeks. Trial participants were recruited starting in August 2017 and the data will be analyzed after the sample size is achieved according to the study design. The flow chart of the study design is shown in Fig. 1. Apart from the time and contents of the designed exercise plan being different between the study group and the control group, relative regulations of the hospital at other times are followed to ensure regular work performance in the hospital and ensure that the treatment of BC patients is not affected in the hospital. All participants will provide informed consent forms before entering the study.

### Participants and recruitment

Individuals who meet the inclusion criteria will be briefly introduced to this study and informed about the benefits as well as possible risks by the clinician. The participants will then be required to fill in consent forms and are free to withdraw from the study at any time without any specific reason, penalty, or loss of benefits.

### Inclusion criteria

Patients with BC phases I–III, who have finished postoperative radiotherapy/chemotherapy within four months to two years will be included.

### Exclusion criteria

Individuals who meet any of the following criteria will be excluded: (1) those with communication or language barriers and inability to complete the questionnaire; (2) those with metastasis, severe deterioration of conditions, or severe mental disorders; (3) those with a history of acute attempted suicide; (4) those with cognitive organic brain lesions and dementia; (5) those who cannot perform exercises of medium exertion and above; and (6) those who cannot use independently or with help social media app (WeChat official account), free-of-charge commercial smartphone apps, and remote video. Patients who meet one or more of the above criteria will be excluded from the study.

### Recruitment strategies

Participants will be recruited from the Department of Breast Surgery, the Second Hospital of Shandong University, Jinan, Shandong. Recruitment is mainly through the doctors’ recommendations, patients publicizing the trial, and the distribution of leaflets. Patients interested in this trial will first be evaluated by physicians. If they meet the criteria and decide to participate, they will be asked to sign the informed consent. They will then be included in the trial for randomization.

### Blinding

After completing the baseline questionnaire and physical fitness tests, the participants will be enrolled and a computer-generated random list generated by SPSS 16.0 will be used for randomization. The random number list is kept strictly confidential by the Data Coordination Committee (DCC) staff. Eligible patients will be randomized to the experimental group or the control group at a 1:1 ratio. The group numbers will be provided in envelopes made from carbonless paper. The envelopes will be kept by a study administrator who will not directly participate in the recruitment or follow-up of any participant and the group numbers will be subsequently disclosed. Different people will enroll participants and assign participants to interventions.

The division results are kept by the study designer until the end of the study to ensure allocation concealment, which means the allocation is bound to both the result evaluator and the data analyzer. The participants and the treatment provider will be instructed not to disclose the allocation to the result evaluator.

### Control group

Participants who have been randomized into the control group will receive normal treatment and examination according to the daily specifications of the hospital. The rehabilitation plan will also be performed according to the regular requirements and postoperative BC rehabilitation-related health education will be provided.

### Intervention group

Basic contents of intervention in the study group include cardiopneumatic endurance training, muscle training, conventional fitness exercises, and postoperative rehabilitation knowledge which pushed combined interventions: (1) cardiopneumatic endurance training: to be performed four times per week, determine the strength of the exercise via rate of perceived exertion (RPE), ask the individuals to complete the target number of steps within the specified time and record the number of steps via a phone step-recording app; (2) muscle training, fitness Qigong exercises: muscle training includes muscle strength, muscle endurance, and muscle function training while fitness Qigong exercise is scheduled after completion of muscle training, practicing certain Qigong Baduanjin fitness routines. Face-to-face remote video guidance on physical exercise rehabilitation is performed three times per week for 30 min each time, including a 5-min warm-up, 20 min of muscle training, and 5 min of relaxation. The primary content of muscle training is endurance training in the first month, strength training in the second month, and muscle function training in the third month. Traditional fitness exercise is scheduled after warming up and relaxing exercises and may include one or more routines each time; (3) postoperative BC rehabilitation knowledge: common knowledge of physical exercise BC rehabilitation pushed regularly by social media apps every day as well as knowledge of simple physical exercise rehabilitation and psychological adjustment relative to BC survivors.

Physical exercise rehabilitation training is performed under the guidance of the participants’ professional physiotherapists. The measures of exercise intervention will be gradually advanced and the strength of exercise will be gradually increased. The side effects and severe adverse events will be recorded and reported to the study designer by physiotherapists; the study designer will then determine further measures to take. In this case, other sports-related exercise interventions will stop, while treatment by medication will continue. If necessary, training will be discontinued according to the reports from the therapists and the consultation results from the BC specialists. We call the roll through every video guide, endurance training, and weekly group meeting to improve adherence to intervention protocols. There are no concomitant or prohibited interventions during this trial.

### Participant timeline

**Fig. 2:**
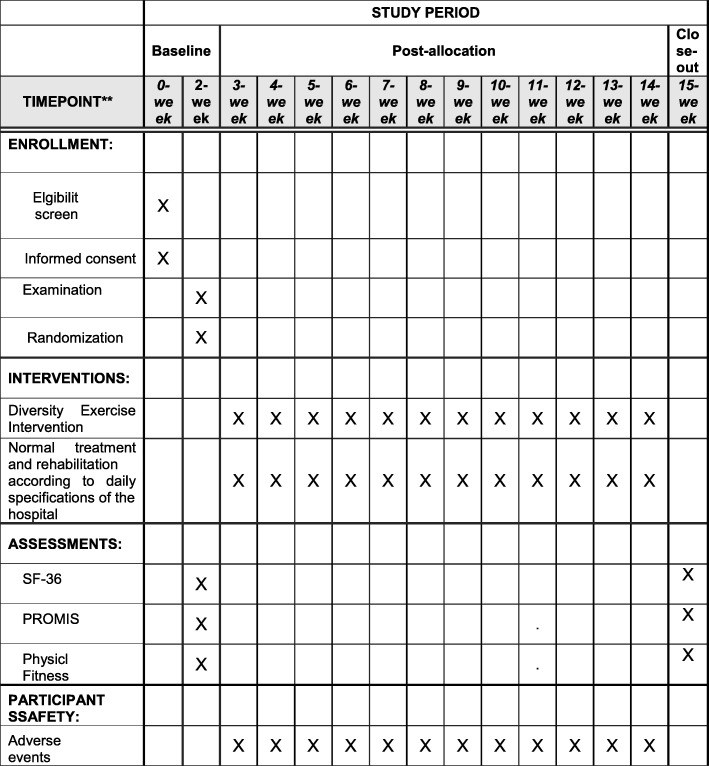
Standard Protocol Items: Recommendations for Interventional Trials (SPIRIT) *diagram* of enrolment, treatment, and assessments over time

The Standard Protocol Items: Recommendations for Interventional Trials (SPIRIT) table in Fig. 2 shows details on the schedule of enrollment, interventions, and assessments.

### Measurements

The primary outcome of the trial will be the MOS 36-item Short Form Health Survey mental component summary scale (SF-36 MCS). The secondary outcomes are other sub-items of the MOS 36-item Short Form Health Survey physical component summary scale (other sub items of SF36) [20, 21], PROMIS, physical fitness, and social cognitive theory. Measurements will be conducted in the same place and by the same tester for both the study group and control group strictly adhering to the precautions and operational rules, to ensure that the measurements are scientific and precise.

### Quality of life (QOL)

1. The MOS 36-item Short Form Health Survey (SF-36)
  SF-36 is a reliable and manageable questionnaire of QOL, which has been extensively applied in the assessment of the health conditions of patients with chronic diseases. SF-36 includes eight dimensions and two summary tables; the score of each dimension is in the range of 1–100 (100 represents the best health condition).
2. The Patient Reported Outcome Measurement Information System [22] (PROMIS) will be used in the evaluation of physical function, anxiety, and depression of the individuals mainly, including physical functions (such as: can you run or do shopping?), anxieties during the past seven days (such as: I feel fearful), frustrating (such as: I feel useless), sleeping disorders (such as: I have trouble falling asleep), and pain disruptions (does pain limit your housekeeping activities?). The options are categorized into five levels from not at all/no/very weak as the first option to always/very much/very good as the fifth option. It has been demonstrated that PROMIS can be applied in the evaluation of the population of clinic patients [23].

### Physical fitness

1. Maximal oxygen uptake: this is determined by an improved Bruce plank test [24] which will be strictly adhered to; the heart rate will be monitored by a Polar watch during the test.
2. Stand-up and sit-down chair test (number of times standing up from the chair within 30 s): these outcomes will be used to evaluate leg strength and endurance of the individual. Test procedure: (i) put an upright chair (or a folding chair) against the wall (for the sake of safety); (ii) the individual sits in the middle of the chair with the right left feet apart, equal to the shoulders (one foot may be placed slightly in front of the other) and both arms are crossed around the waist and close to the chest; (iii) during the test, the individual should stand up completely and then sit down completely; (iv) the number of times that the individual stands up from and sits down in the chair within 30 s is recorded; (v) for safety purposes, or when necessary, the individual may use her arms for assistance.
3. Number of arm raises (30 s with a dumbbell of 5 lbs or 2.3 kg): this outcome is used to evaluate the strength and endurance of the upper extremities. Test procedure: (i) a 2.3 kg (5 lbs) dumbbell is used; (ii) put an upright chair (or a folding chair) against the wall (for the sake of safety); (iii) the individual sits close to the side of chair if she has a stronger arm on that side; (iv) the stronger arm holds the dumbbell with the arm naturally hanging straight down over one side of the chair while the body takes a push-up position; (v) the non-moving arm is fixed close to the body so that only the moving arm is moving; (vi) bend and lift the arm to its maximum extent when gradually rotating to the outward position with the palm facing up; (vii) the arm returns to the push-up position and is placed on one side of the chair; (viii) the number of times that the arm bends and lifts is recorded; (ix) precautions include making sure the arm is completely bent and the elbow is completely straightened—it is very important that the upper extremities are stable without swaying.
4. Percentage of body fat [24]: measuring the (i) upper arm (triceps)—vertical direction of skin folds; both arms fall naturally on both sides of the body, measure at the midpoint of the straight line connecting the acromion and the olecranon of the posterior upper arm; (ii) anterior superior iliac spine—oblique direction of the skin folds; measure at the axillary line above the anterior superior iliac spine and follow the natural wing of the ilium; (iii) abdominal skin folds—vertical direction of the skin folds, at 2 cm from the right side of umbilicus. Measure the thickness of the skin folds at these three points, calculate the body density with the three-point method according to the female skin fold measurement and calculation formula, and then calculate the individual’s percentage of body fat.
5. Height, body weight, and flexibility tests.

### Social cognitive indicator

The participant’s social cognitive belief will be evaluated by standard self-report questionnaire [25] including: sport exercises: social support questionnaire; sport exercises: obstruction questionnaire; sport exercises: expected outcome questionnaire; sport exercises: self-efficiency questionnaire. For example: sport exercises: social support questionnaire is to survey whether your family and friends have completed the following items during the past 30 days, including five social support questions (encourage you to exercise, discuss the disadvantages of not exercising, remind you to exercise, share some methods of exercise with you, do exercises together with you). The options include “almost none,” “once,” “sometimes,” “often,” and “always” in sequence. These questionnaires have been subject to normal distribution validation and content validity analysis in previous literature.

### Sample size

A total of 52 participants (26 in each group) completing follow-up will provide 90% power (α = 0.05) to detect 9.5 points [26] in SF-36 MCS assuming a standard deviation of 11.6 points [27]. Assuming a 15% drop-out rate between baseline and follow-up, 60 participants will be enrolled in the study.

### Data collection and management

To ensure the accuracy of outcome assessments and data collection, all of the physicians, nurses, and research assistants will attend a training workshop before the start of the trial. All attendees will be provided with a protocol and standard operation procedures and will discuss the topics they may feel confused about until everyone is totally clear about the procedures. We do not have plans to promote participant retention and complete follow-up.

After screening and signing of informed consent and end-of-study measurements, the SF-36, physical fitness, and social cognitive theory will be carefully recorded. Monitoring will be performed by personnel independent of the investigators and the sponsor and will consist of checking all informed consent forms and completeness of all data and source data verification of patients. Data are collected and recorded on a standard report form; when the visit is completed, all the recorded data will be entered into the web-based data system by the double-entry method. All errors will need to be corrected by crossing out, with the researcher’s signature and date. Any participant may quit the study at any time for any reason. If any patients want to quit, clinicians will ask whether they agree to finish the follow-up according to the study schedule. All patients who quit and are lost to follow-up will be recorded. If our study fails to recruit 50% of patients within six months, we will stop the study. The process will be independent from investigators and the sponsor.

### Statistical analyses

The study results will undergo statistical analysis with SPSS software version 19.0. Comparisons between groups will be an analysis of covariance (ANCOVA) with treatment and baseline entered in the model. Estimate, 95% confidence intervals (CIs), and *p* values will be reported. The analysis population will adopt Intent to treat (ITT) and ignoring missing data.

### Ethical approval and dissemination

This study follows the principles of the Declaration of Helsinki (Version Edinburgh 2000). Before participating in this research, every potential participant should be informed of the detailed study information, while participants can ask questions by phone, e-mail, and face to face. Only patients who sign the informed consent form will be included. This study has been approved by the Ethics Committee of the Second Hospital of Shandong University (KYLL-2017(KJ)P-0003). Personal information about potential and enrolled participants will be collected and stored separately from medical records and will be only accessible for the assessors who contact the participants for data assessment. In case of an unexpected serious adverse event (e.g. life-threatening event or permanent damage) over the course of the study, we will report the serious adverse event to the local Ethics Committee. The Ethics Committee and the study team will then decide in accordance, in the best interests of the patient, if the study procedures will be continued or terminated. The results of this study will be disseminated by presenting at international conferences and publication in peer-reviewed journals. Participants will learn about the results of the study.

The principal investigator is responsible for publication of the results of this study in whole or in part. Neither the complete nor any part of the results of the study carried out under this protocol, nor any of the information provided by the sponsor for the purposes of performing the study, will be published or passed on to any third party without the consent of the study sponsor. The study designer will have access to the final trial dataset. Any investigator involved with this study cannot access the trial data. Data sharing statement is as follows: no later than three years after the collection of the one-year post-randomization interviews, we will deliver a completely deidentified dataset to an appropriate data archive for sharing purposes.

## Discussion

There have been studies, both domestic and abroad, on physical exercise interventions with social media apps or smartphone apps in postoperative BC patients. However, as far as we know, the ways of intervention and measures of practice in these studies are relatively simple and few combined intervention studies have been conducted. Therefore, we propose this protocol of combined physical exercise intervention based on Internet and social media apps and plan to validate the feasibility and effectiveness of this protocol in BC rehabilitation via empirical research. If the expected results are achieved in this study, measures and methods of BC rehabilitation will be enriched. Meanwhile, it will also provide support for physical exercise interventions for other diseases, promoting the development of prevention and rehabilitation through physical exercise for patients with chronic diseases, who may also experience social and economic benefits as a result.

In this study, our objective is to perform a clinical trial with a relatively high standard of methods. The protocol of this trial, implementation, and reports will comply with the recommendations in Consolidate Standards of Reporting Trial (CONSORT). A Standard Protocol Items: Recommendations for Interventional Trials (SPIRIT) [28] diagram is provided in Additional file 1.

### Trial status

At the time of manuscript submission, recruitment for the study is underway but has not been completed.

## Additional file

Additional file 1: SPIRIT 2013 checklist: Recommended items to address in a clinical trial protocol and related documents. (DOC 142 kb)

## Abbreviations

BC: Breast cancer
CONSORT: Consolidated Standards of Reporting Trials
DCC: Data Coordination Committee
QOL: Quality of life
RPE: Rate of perceived exertion
SF-36: 36-item Short Form Health Survey
