# Supplementary material for: The effects of combined exercise intervention based on Internet and social media software for postoperative patients with breast cancer: study protocol for a randomized controlled trial
Source: Trials. 2018 Sep 6;19:477. doi: 10.1186/s13063-018-2857-3 (PMC6127941; doi:10.1186/s13063-018-2857-3)
Supplement: Supplementary file 1 — SPIRIT 2013 checklist: Recommended items to address in a clinical trial protocol and related documents. (DOC 142 kb) [file 13063_2018_2857_MOESM1_ESM.doc]

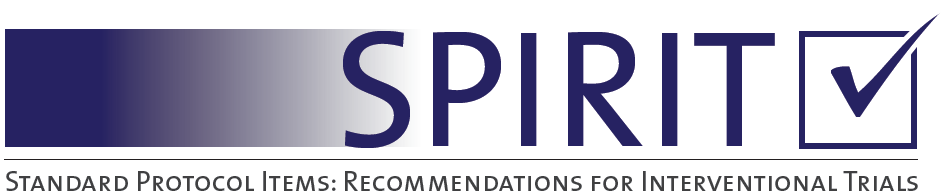


SPIRIT 2013 Checklist: Recommended items to address in a clinical trial protocol and related documents* The paper have been revised by English language editing.

| Section/item | Item No | Description | Addressed on page number |
| --- | --- | --- | --- |
| **Administrative information** | | |  |
| Title | 1 | Descriptive title identifying the study design, population, interventions, and, if applicable, trial acronym | _______1__ |
| Trial registration | 2a | Trial identifier and registry name. If not yet registered, name of intended registry | _______1______ |
|  | 2b | All items from the World Health Organization Trial Registration Data Set | ______1_______ |
| Protocol version | 3 | Date and version identifier | ______N/A______ |
| Funding | 4 | Sources and types of financial, material, and other support | ______9_______ |
| Roles and responsibilities | 5a | Names, affiliations, and roles of protocol contributors | _____9-10_______ |
|  | 5b | Name and contact information for the trial sponsor | ______9-10_______ |
|  | 5c | Role of study sponsor and funders, if any, in study design; collection, management, analysis, and interpretation of data; writing of the report; and the decision to submit the report for publication, including whether they will have ultimate authority over any of these activities | _____9______  AE: this must be specified as it is not made explicit in the attached funding letter  We have made explicit in the attached funding letter. |
|  | 5d | Composition, roles, and responsibilities of the coordinating centre, steering committee, endpoint adjudication committee, data management team, and other individuals or groups overseeing the trial, if applicable (see Item 21a for data monitoring committee) | ____N/A________ |
| Introduction |  |  |  |
| Background and rationale | 6a | Description of research question and justification for undertaking the trial, including summary of relevant studies (published and unpublished) examining benefits and harms for each intervention | _______2-3____ |
|  | 6b | Explanation for choice of comparators | _____N/A_______ |
| Objectives | 7 | Specific objectives or hypotheses | _______3______ |
| Trial design | 8 | Description of trial design including type of trial (eg, parallel group, crossover, factorial, single group), allocation ratio, and framework (eg, superiority, equivalence, noninferiority, exploratory) | ___ _3______ |
| Methods: Participants, interventions, and outcomes | | |  |
| Study setting | 9 | Description of study settings (eg, community clinic, academic hospital) and list of countries where data will be collected. Reference to where list of study sites can be obtained | _______3______ |
| Eligibility criteria | 10 | Inclusion and exclusion criteria for participants. If applicable, eligibility criteria for study centres and individuals who will perform the interventions (eg, surgeons, psychotherapists) | ________3-4_____ |
| Interventions | 11a | Interventions for each group with sufficient detail to allow replication, including how and when they will be administered | ________4-5_____ |
|  | 11b | Criteria for discontinuing or modifying allocated interventions for a given trial participant (eg, drug dose change in response to harms, participant request, or improving/worsening disease) | _______N/A_______ |
|  | 11c | Strategies to improve adherence to intervention protocols, and any procedures for monitoring adherence (eg, drug tablet return, laboratory tests) | ______4_______ AE: I could not find this explained in page 4. Can you please state the sentence and/or amend the manuscript accordingly?  We call the roll through every video guide, endurance training and weekly group meeting to improve adherence to intervention protocols. |
|  | 11d | Relevant concomitant care and interventions that are permitted or prohibited during the trial | _______N/A______ AE: I have found the explanation of experimental group but not concomitant/prohibited interventions. Can you please state the sentence and/or amend the manuscript accordingly?  There are no concomitant or prohibited interventions during this trial. |
| Outcomes | 12 | Primary, secondary, and other outcomes, including the specific measurement variable (eg, systolic blood pressure), analysis metric (eg, change from baseline, final value, time to event), method of aggregation (eg, median, proportion), and time point for each outcome. Explanation of the clinical relevance of chosen efficacy and harm outcomes is strongly recommended | _____5-7_______  AE: I would suggest to have only one primary end point (e.g. SF36 PCS or MCS) otherwise the sample size should be taken in account both primary end points. The other will be secondary end points. It should be made explicit at which timepoint  We have modified. |
| Participant timeline | 13 | Time schedule of enrolment, interventions (including any run-ins and washouts), assessments, and visits for participants. A schematic diagram is highly recommended (see Figure) | ____5________ AE: I would say this is applicable otherwise procedures could not be ascertained. For example: how many visits the patients will attend? At which timepoint endpoints are collected? When it will be the end of study visit? It looks like there is only baseline and end of study but please confirm. I would suggest you read the examples within the SPIRIT guidelines  We have read the examples within the SPIRIT guidelines and added time schedule. |
| Sample size | 14 | Estimated number of participants needed to achieve study objectives and how it was determined, including clinical and statistical assumptions supporting any sample size calculations | ______7_______  AE: the sample size does not include the expected difference in one of the component of the SF36 (e.g. SF36 PCS; as stated I suggest to only have only one primary outcome otherwise sample size should take in account all primary end points) and SD. Please revise.  We have revised in the manuscript. |
| Recruitment | 15 | Strategies for achieving adequate participant enrolment to reach target sample size | ________4_____ AE: can you please make this explicit? I have read you expect a 15% loss to follow up however I could not find which strategies you will implement in order to achieve the stated sample size and how to minimize drop out.  The recruitment is mainly through the doctors' recommendations, the patients publicizing the tria and the distribution of leaflets. |
| **Methods: Assignment of interventions (for controlled trials)** | | |  |
| Allocation: |  |  |  |
| Sequence generation | 16a | Method of generating the allocation sequence (eg, computer-generated random numbers), and list of any factors for stratification. To reduce predictability of a random sequence, details of any planned restriction (eg, blocking) should be provided in a separate document that is unavailable to those who enrol participants or assign interventions | _____7_______ AE: it is not made explicit how the list is generated (e.g. computer software? Website?). It is not made explicit which method will be used (E.g. simple randomization). Please beware on disclosing details of the methods on the protocol.  We have bewared on disclosing details of the methods on the protocol. |
| Allocation concealment mechanism | 16b | Mechanism of implementing the allocation sequence (eg, central telephone; sequentially numbered, opaque, sealed envelopes), describing any steps to conceal the sequence until interventions are assigned | ___4________ AE: I could be wrong but this is not made explicit.  The group numbers will be provided in envelopes made from carbonless paper. The envelopes will be kept by a study administrator who will not directly participate in the recruitment or follow-up of any participant, and the group numbers will be subsequently disclosed. |
| Implementation | 16c | Who will generate the allocation sequence, who will enrol participants, and who will assign participants to interventions | ____4_______ AE: I could be wrong but this is not made explicit.  The random number list is kept strictly confidential by the Data Coordination Committee (DCC) staff. Eligible patients will be randomized to the experimental group or the control group at a 1:1 ratio. Different people will enroll participants and assign participants to interventions. |
| Blinding (masking) | 17a | Who will be blinded after assignment to interventions (eg, trial participants, care providers, outcome assessors, data analysts), and how | ______4______  AE: I would suggest to reword the sentence “It is suggested that the subjects and the treatment provider won’t  disclose the allocation to the result evaluator.” to “the subjects and the treatment provider will be instructed not to disclose the allocation to the result evaluator.”  We have revised in the manuscript. |
|  | 17b | If blinded, circumstances under which unblinding is permissible, and procedure for revealing a participant’s allocated intervention during the trial | ____4-5________ |
| **Methods: Data collection, management, and analysis** | | |  |
| Data collection methods | 18a | Plans for assessment and collection of outcome, baseline, and other trial data, including any related processes to promote data quality (eg, duplicate measurements, training of assessors) and a description of study instruments (eg, questionnaires, laboratory tests) along with their reliability and validity, if known. Reference to where data collection forms can be found, if not in the protocol | _______7______ |
|  | 18b | Plans to promote participant retention and complete follow-up, including list of any outcome data to be collected for participants who discontinue or deviate from intervention protocols | _______7_______ AE: In my opinion this is applicable. You can also state in the protocol there will be no such plans  We don’t have plans to promote participant retention and complete follow-up |
| Data management | 19 | Plans for data entry, coding, security, and storage, including any related processes to promote data quality (eg, double data entry; range checks for data values). Reference to where details of data management procedures can be found, if not in the protocol | _______7______ AE: I could be wrong but I could not find it.  Monitoring will be performed by personnel independent of the investigators and the sponsor and will consist of checking all informed consent forms and completeness of all data and source data verification of patients. Data are collected and recorded on a standard report form, and when the visit is completed, all of the recorded data will be entered into the web-based data system by the double-entry method. All errors will need to be corrected by crossing out, with the researcher’s signature and date. |
| Statistical methods | 20a | Statistical methods for analysing primary and secondary outcomes. Reference to where other details of the statistical analysis plan can be found, if not in the protocol | _______7______ AE: I would suggest analysis on primary outcome (e.g. SF36 PCS) will be an Analysis of Covariance (ANCOVA) with treatment and baseline entered in the model. Estimate, 95% Confidence intervals (CIs) and p values will be reported. A similar strategy will be used for other continuous end points. Please do not perform “within comparisons” in parallel randomized trials. An article discusses the issue (<https://www.bmj.com/content/342/bmj.d561.full.print>). Categorical data can be analysed via a logistic regression reporting estimates, 95%Cis and p values.  We have revised in the manuscript. |
|  | 20b | Methods for any additional analyses (eg, subgroup and adjusted analyses) | ________7_____ AE: Please be advised that “within groups” are not considered subgroup analyses. These are usually performed on categorization of baseline data (e.g. analysis on male vs female or analysis with and without a comorbidity). I would suggest ANCOVA and logistic regression as adjusted analyses.  We have revised in the manuscript. |
|  | 20c | Definition of analysis population relating to protocol non-adherence (eg, as randomised analysis), and any statistical methods to handle missing data (eg, multiple imputation) | _______7_____ AE: please report population (e.g. Intention To Treat or ITT) and methods on how to handle missing data (ignoring missing data could be an option)  Analysis population will adopt ITT and ignoring missing data. |
| **Methods: Monitoring** | | |  |
| Data monitoring | 21a | Composition of data monitoring committee (DMC); summary of its role and reporting structure; statement of whether it is independent from the sponsor and competing interests; and reference to where further details about its charter can be found, if not in the protocol. Alternatively, an explanation of why a DMC is not needed | _______N/A________ |
|  | 21b | Description of any interim analyses and stopping guidelines, including who will have access to these interim results and make the final decision to terminate the trial | _______7______ AE: I could not find it. Beware that interim analyses could affect the statistical type I error of atrial. I would suggest to use “non formal” rules for stopping the trials (e.g. stopping the trial if this does not recruit xx patients after some time have elapsed) or “simple” formal rules like conditional power for stopping for futility (see Sully2014 <https://trialsjournal.biomedcentral.com/articles/10.1186/1745-6215-15-61)>  If our study fails to recruit 50% of patients within 6 months, we will stop the study. |
| Harms | 22 | Plans for collecting, assessing, reporting, and managing solicited and spontaneously reported adverse events and other unintended effects of trial interventions or trial conduct | __________N/A____ |
| Auditing | 23 | Frequency and procedures for auditing trial conduct, if any, and whether the process will be independent from investigators and the sponsor | _______7______ AE: I could be wrong but I could not find it.  The process will be independent from investigators and the sponsor. |
| Ethics and dissemination | | |  |
| Research ethics approval | 24 | Plans for seeking research ethics committee/institutional review board (REC/IRB) approval | ______7-8_______ |
| Protocol amendments | 25 | Plans for communicating important protocol modifications (eg, changes to eligibility criteria, outcomes, analyses) to relevant parties (eg, investigators, REC/IRBs, trial participants, trial registries, journals, regulators) | ________N/A____ |
| Consent or assent | 26a | Who will obtain informed consent or assent from potential trial participants or authorised surrogates, and how (see Item 32) | _______7______ |
|  | 26b | Additional consent provisions for collection and use of participant data and biological specimens in ancillary studies, if applicable | _________N/A______ |
| Confidentiality | 27 | How personal information about potential and enrolled participants will be collected, shared, and maintained in order to protect confidentiality before, during, and after the trial | _______8_____ AE: I could be wrong but I could not find it.  Neither the complete nor any part of the results of the study carried out under this protocol, nor any of the information provided by the sponsor for the purposes of performing the study, will be published or passed on to any third party without the consent of the study sponsor. |
| Declaration of interests | 28 | Financial and other competing interests for principal investigators for the overall trial and each study site | _______9______ |
| Access to data | 29 | Statement of who will have access to the final trial dataset, and disclosure of contractual agreements that limit such access for investigators | __________98_____ AE: please make explicit this point especially if other investigators can ask for accessing the trial data.  The study of designer will have access to the final trial dataset. Any investigator involved with this study cannot access the trial data. |
| Ancillary and post-trial care | 30 | Provisions, if any, for ancillary and post-trial care, and for compensation to those who suffer harm from trial participation | _________N/A______ |
| Dissemination policy | 31a | Plans for investigators and sponsor to communicate trial results to participants, healthcare professionals, the public, and other relevant groups (eg, via publication, reporting in results databases, or other data sharing arrangements), including any publication restrictions | _________8____ |
|  | 31b | Authorship eligibility guidelines and any intended use of professional writers | _________N/A______ |
|  | 31c | Plans, if any, for granting public access to the full protocol, participant-level dataset, and statistical code | _______8______ AE: please make explicit whether you will disclose participant-level dataset, and statistical code  Data sharing statement No later than 3 years after the collection of the 1-year postrandomisation interviews, we will deliver a completely deidentified data set to an appropriate data archive for sharing purposes. |
| Appendices |  |  |  |
| Informed consent materials | 32 | Model consent form and other related documentation given to participants and authorised surrogates | _____N/A_______ |
| Biological specimens | 33 | Plans for collection, laboratory evaluation, and storage of biological specimens for genetic or molecular analysis in the current trial and for future use in ancillary studies, if applicable | _____N/A_______ |

*It is strongly recommended that this checklist be read in conjunction with the SPIRIT 2013 Explanation & Elaboration for important clarification on the items. Amendments to the protocol should be tracked and dated. The SPIRIT checklist is copyrighted by the SPIRIT Group under the Creative Commons “[Attribution-NonCommercial-NoDerivs 3.0 Unported](http://www.creativecommons.org/licenses/by-nc-nd/3.0/)” license.
